# How might enhanced interprofessional collaboration between primary care physicians and registered dietitian nutritionists impact clinical outcomes related to obesity and associated illnesses? A commentary

**DOI:** 10.1017/S1368980022002518

**Published:** 2023-03

**Authors:** Leah Qubty, Kristen Hicks-Roof

**Affiliations:** 1 Department of Applied Health Sciences, Bethel University, St. Paul, MN, USA; 2 Department of Nutrition & Dietetics, Brooks College of Health, University of North Florida, Jacksonville, FL 32224, USA

**Keywords:** Physician nutrition education, Referral, Nutrition education, Interprofessional collaboration, Chronic-related diseases

## Abstract

The unsettling worldwide prevalence of obesity and obesity-related illnesses seems to be well-understood. What seems to be lacking, however, is a strategy of how best to fix the issue. Meagre nutrition content in medical school curricula may contribute to limited primary care physician (PCP) knowledge of the role nutrition has in health ailments and the understanding of a registered dietitian nutritionist (RDN) scope of practice. In USA, RDN are health care practitioners specialised in nutrition and who are experts in treating obesity and obesity-related illnesses. An increased RDN involvement in patient care has been shown to promote improvements in nutrition-related clinical outcomes. Therefore, enhanced collaboration between PCP and RDN has the potential to ameliorate the obesity epidemic. Tactics to promote collaboration may include enhancing nutrition education in medical school and providing nutrition-focussed continuing education for practicing physicians. The seriousness of the obesity epidemic underscores the need for interprofessional collaboration between PCP and allied health professionals who are uniquely trained to address obesity and obesity-related illnesses.

The devastating effects of obesity and obesity-related illnesses on the health of the global population no longer seem to shock readers. In 2016, 650 million adults and 381 million children were obese worldwide^(1)^. Fortunately, awareness of this problem has improved^(2)^. Unfortunately, in many ways, it does not seem as though we are closer to a solution. US overweight and obesity statistics from the Centers for Disease Control and Prevention abound in the literature, and the Centers for Disease Control and Prevention overweight and obesity incidence maps seem to frequently appear in public health campaigns. Essentially, we know, as a scientific community, that obesity and obesity-related chronic diseases are a problem. What seems to be missing, however, is a consensus on how to fix it^(3)^.

The Global Burden of Disease (GBD) study assessed the global disease burden related to high BMI^(3)^. The results of the GBD study mirrored the aforementioned Centers for Disease Control and Prevention statistics and underscored the serious nature of the high worldwide prevalence of elevated BMI. In fact, the GBD study, which incorporated the information from 68·5 million individuals, found that, in 2015, overweight and obesity were responsible for 4 million deaths and 120 million disability-adjusted-life years worldwide^(3)^. Sedentary lifestyle is often the scapegoat for rising obesity rates. Interestingly, the investigators of the GBD study note that although changes in the built environment such as increased urbanisation promote decreased physical activity, they are less likely contributors to the obesity epidemic than dietary factors as they often preceded rises in obesity^(3)^. An earlier GBD study also noted that five of the seven leading causes of premature death and years lived with disability are directly related to diet. The role of dietary factors in poor clinical outcomes might be especially obvious in the USA. Currently, the USA spends more than any other country on health care; the discrepancy is largely related to obesity and related illnesses^(4)^.

Statistical information on the incidence of overweight, obesity and obesity-related illnesses is extremely useful in helping to quantify the problem. However, these statistics do little in the way of helping to construct a solution. As we aim to move towards a solution, it seems appropriate to ascertain which healthcare providers patients currently seek out for health advice, identify the allied health professional specifically trained to address obesity and related illnesses and analyse interprofessional collaboration. We aim to assist the public health community in the US move towards identifying a uniform solution to the obesity epidemic.

## Discussion

### Nutrition training

The National Academy of Sciences recommends 25 h of nutrition education be provided during medical school in the USA^(5)^. However, it has been shown that 71 % failed to provide this recommended 25 h^(6)^. Moreover, the small amount of nutrition education is largely provided in the early years of medical school and has little connection to common diseases^(7)^. The issue of meagre nutrition education within medical school curricula is not a new one. The National Nutrition Monitoring and Related Research Act of 1990 noted the inconsistency between national public health goals and attention to nutrition-related matters in medical school curricula in the USA^(8)^, and some sources suggest this issue dates as far back as 1950^(9)^. Poor nutrition education in medical school curricula extends beyond US medical schools. When medical schools in the USA, the Middle East, Australia, Europe and Africa were assessed, nutrition education in medical school curricula was lacking in each country^(10)^. Not only was nutrition education lacking but also it appeared to have a direct negative impact on medical student’s nutrition knowledge and confidence to address nutrition with their patient population^(10)^.

According to Aggarwal *et al.*, when practicing physicians were surveyed, 22 % remembered receiving zero nutrition education throughout medical school^(11)^. Moreover, among the same physicians who *did* recall receiving nutrition education in medical school, 35 % reported that nutrition education was limited to a single lecture^(11)^. Medical students’ perceptions of nutrition education curricula in medical school reflect the sparse amount of nutrition training offered. Medical students felt as though nutrition was only mildly incorporated into their coursework, and they felt the nutrition education provided in medical school was inadequate^(5)^. Survey data from internal medicine residents reported that only 14 % felt prepared to address nutrition-related issues with their patients^(12)^. Additionally, 94 % felt obligated to discuss nutrition with their patients^(12)^. Feelings of unpreparedness among medical residents are likely due to an overall lack of nutrition training in medical schools in the USA, which is a repercussion from little to no accreditation standards focussed on nutrition^(13)^.

In contrast, registered dietitian nutritionists (RDN) in the USA undergo rigorous, standardised, didactic training in the field of nutrition and dietetics and are well-equipped to address obesity and obesity-related illnesses with patients^(14)^. Moreover, RDN are educated in behaviour change theories and motivational interviewing, both of which help the RDN establish how best to help the patient achieve their nutrition goals^(15)^. Healthy lifestyle approaches effectively reduce BMI among individuals with obesity^(16)^. However, the implementation of these lifestyle interventions is the main barrier for most individuals^(16)^. While 14 % of physicians feel prepared to address nutritionally pertinent topics with their patients^(12)^, 92 % of RDN feel they are the most qualified professionals to assist patients with weight loss^(17)^. These data suggest that if patients with obesity or obesity-related illnesses were appropriately referred to an RDN, their chances of clinical success would improve greatly.

Considering the seriousness of obesity and obesity-related illness prevalence in the USA, it is problematic that resident physicians feel relatively unprepared to provide nutrition counselling to their patients^(12)^. Hicks *et al.* highlight the discrepancy in medical school nutrition education and consumer confidence in physician nutrition knowledge^(18)^. According to the Centers for Disease Control and Prevention, 51·2 % of all healthcare visits in the USA are with a PCP^(19)^. Moreover, 64 % of Americans feel physicians are well-equipped to provide nutrition recommendations^(20)^. These data suggest primary care physicians (PCP) have an unique opportunity to direct patients with nutritionally pertinent health ailments towards a healthcare professional trained in nutrition (e.g. a RDN).

### Current interprofessional collaboration

The fact that physicians receive minimal nutrition education^(6)^, and consequently feel ill-equipped to provide patients with nutrition recommendations^(12)^ suggests physicians might rely heavily on RDN referrals. Yet, data on patient access to RDN services appear extremely limited. Moreover, physicians feel obligated to address nutrition^(12)^ and feel as though clinical outcomes would improve greatly by incorporating nutrition into patient care^(11)^. These data suggest insight into the referral process between PCP and US RDN is warranted.

The confidence RDN feel to address obesity and obesity-related illnesses is justified. When RDN were incorporated into the offices of PCP, they effectively improved clinical outcomes related to nutrition^(21)^. However, PCP referrals to nutrition services are lacking in some settings. For example, RDN currently provide weight loss counselling through a Veterans Health Administration weight loss programme^(22)^. Less than 10 % of eligible patients are referred to the programme. When referring PCP were interviewed, poor anticipated clinical outcomes were identified as a major referral barrier^(22)^. The results of this study suggest PCP may have poor insight into the RDN knowledge base and scope of practice.

A recent study conducted by Fitzpatrick *et al.* assessed whether an alert in the medical record prompted physicians to refer patients with a BMI indicative of obesity to an RDN^(23)^. The researchers concluded the medical record alert did not effectively improve RDN referrals^(23)^. These data suggest the intervention to improve physician referrals to RDN must be more involved. A study assessing referrals to RDN by physicians working in the primary care setting found that a misunderstanding of the role nutrition education plays in chronic disease management was the foremost reason PCP did not refer their patients to a RDN^(24)^. Therefore, it appears the lack of nutrition education offered in medical school is not only having a negative impact on the amount of nutrition education physicians are offering to their patients, but also is having a negative impact on referrals to RDNs as well. A lack of PCP knowledge about the knowledge base and scope of practice of the RDN appears to be barrier to RDN referrals^(25)^.

### Improving interprofessional collaboration: possible solutions

Improving nutrition education for physicians might facilitate interprofessional collaboration between PCP and RDN. Interventions to bolster medical school nutrition curricula have a positive impact on the nutrition knowledge of medical students and physicians^(26)^, which is translating to positive clinical outcomes^(26)^. RDN are qualified educators in this context and should play an integral role in educating physicians throughout their training and practice^(27)^. Not only are RDN qualified educators in this context but also exposure to an RDN in medical school or through continuing education might increase physicians’ knowledge about the RDN scope of practice and knowledge base, and therefore has the potential to improve physician referrals to RDN.

Several medical schools in the USA have recognised the lack of nutrition education in their curricula and have attempted to ameliorate the issue. For example, the University of Nevada School of Medicine integrated nutrition-based curricula into their medical school^(28)^. Upon review of these curricula, they found that they were unique among medical schools in the USA and felt that positivity and being proactive facilitated nutrition education integration^(28)^. The University of Pennsylvania also attempted to independently integrate nutrition information into their medical school curricula with success^(29)^. Prior to the integration of nutrition curricula, 80 % of medical students at the University of Pennsylvania thought nutrition education was inadequate. After the nutrition curricula had been integrated, only 10 % of medical students felt that way^(29)^. When integrating nutrition into medical school curricula, foreseen issues include limited faculty time and ‘issues of control and inertia’^(30)^. Effective strategies include a ‘forward-thinking attitude’ of the institution and ‘creativity and innovative strategies’ used by nutrition faculty and administrative staff^(31)^.

Although nutrition education within medical school curricula appears to be an effective way to improve the nutrition knowledge of physicians, continuing medical education initiatives should also be considered to support physician nutrition acumen. A recent study by Hicks and Murano assessed whether an online continuing medical education course created by an RDN and physician PCP effectively improved nutrition knowledge relating to type 2 diabetes among practicing physicians. This course effectively improved nutrition knowledge relating to type 2 diabetes management among physicians and awareness of the role of the RDN^(32)^. Virtual seminars may be an effective tool to disseminate nutrition information to medical students as well^(33)^. However, in the context of medical school and continuing medical educations, challenges exist with virtual learning, such as a lack of audience engagement^(34)^.

## Conclusion

Although the high prevalence of obesity and obesity-related illnesses is not recent news to the US scientific community, the large role dietary factors are playing in this epidemic might be. However, there is hope—US RDN are available and trained to help. RDN are nutrition professionals who undergo rigorous, didactic training in dietetics and who are trained to address nutritionally relevant health ailments. The data presented in this manuscript suggest a solution to the obesity epidemic might lie in a more streamline referral process between PCP and dietitians. Patient access to RDN might be bolstered through improved nutrition education in medical school or through continuing education. The large role dietary factors have in the obesity epidemic stresses the need to enlist RDN in the fight against it.
